# PSMA-Targeted Liposomes for Therapeutic Delivery into a Prostate Cancer Cell Model

**DOI:** 10.3390/pharmaceutics18091150

**Published:** 2026-09-14

**Authors:** Miguel Ferreira, Neelum Aziz Yousafzai, Lital Ben Naim, Bahar Ataeinia, Umar Mahmood, Pedram Heidari

**Affiliations:** Division of Nuclear Medicine and Molecular Imaging, Department of Radiology, Massachusetts General Hospital, Harvard Medical School, 55 Fruit St. White 427, Boston, MA 02114, USAnyousafzai@mgh.harvard.edu (N.A.Y.); lbennaim@mgh.harvard.edu (L.B.N.); um1@stanford.edu (U.M.)

**Keywords:** prostate cancer, PSMA targeting, lipid nanoparticles (LIPOs), talazoparib, combination therapy, drug delivery, preclinical evaluation

## Abstract

**Background:** Prostate cancer (PCa) remains a significant health challenge, requiring innovative treatments. Here, we evaluated the efficacy of Talazoparib-loaded liposomes incorporating a targeting moiety, in combination with BI 2536, in PCa. This study develops lipid nanoparticles (LIPOs) targeting prostate-specific membrane antigen (PSMA) for better drug delivery. **Methods:** Two new PSMA-targeting agents, DUPA-DSPE and DUPA-PEG-DSPE, were created and incorporated into LIPOs via thin-film hydration. LIPOs’ size and morphology were characterized using Dynamic light scattering, and structural analysis was done via TEM imaging. In vitro cytotoxicity and cell viability were assessed using an MTT assay, while in vivo radioactivity was evaluated by PET imaging, and radioactive activity was quantified using a γ-counter. **Results:** These LIPOs demonstrated excellent stability and specific binding to PSMA-expressing prostate cancer cells in vitro. In vivo, mouse model studies showed that PSMA-targeted LIPOs accumulated preferentially at tumor sites. Talazoparib, a PARP inhibitor with low solubility and cytotoxicity, has limited use in PCa. **Conclusions:** These findings provide proof-of-concept evidence that PSMA-targeted DUPA-PEG LIPOs can facilitate Talazoparib delivery and, particularly in combination with BI 2536, may enhance antitumor activity in PCa models. Further studies with expanded control groups and larger cohorts are warranted to validate the therapeutic efficacy of this combination strategy and support its further development for PCa treatment.

## 1. Introduction

Prostate cancer (PCa) poses significant health and socioeconomic burdens [1]. The National Cancer Institute anticipates a 27% rise in PCa diagnosis and treatment costs in the United States, from $21.4 billion in 2020 to $27.2 billion by 2030 [2]. Early-stage PCa is typically managed through radical prostatectomy (RP) or external radiotherapy (RT). At the same time, advanced or metastatic cases may require chemical castration, often progressing to metastatic castration-resistant prostate cancer (mCRPC), characterized by high aggressiveness and mortality rates. Treatment modalities are typically tailored to the stage of the disease, the clinical context, and the availability of treatment options. Active surveillance (AS), RP, or RT are initial management options for low-risk patients [3]. Although AS may seem cost-effective due to the potential for metastatic progression, RP and RT show marginal decreases in survival rates, attributed to delayed or prevented treatment complications. In intermediate- to high-risk PCa, RT combined with androgen deprivation therapy (ADT) and RP emerges as a cost-effective option, with RT particularly beneficial for older patients [4,5].

Precise tumor targeting aimed at specific cancer biomarkers for effective drug delivery potentially minimizes the side effects associated with normal tissue damage. In PCa, the highly expressed transmembrane glycoprotein prostate-specific membrane antigen (PSMA) is a crucial target for diagnosis and treatment [6]. PSMA exhibits exceptional tissue specificity to PCa, especially in poorly differentiated, metastatic, and castration-resistant tissues, making it an indispensable tool for PCa management [7,8]. Utilizing PSMA-targeted small molecules such as PSMA-I&T, PSMA-1007, PSMA-617, PSMA-11, DCF-PyL, and rhPSMA-7.3 has shown promising results in PCa imaging and therapy due to their low molecular weight, tissue penetration, rapid blood clearance, and scalability [9,10]. Nanoplatforms (NPs), including metal nanoparticles, polymer-drug conjugates, micelles, liposomes, and dendrimers, offer advantages over conventional formulations by increasing drug solubility, mitigating cytotoxicity, and improving drug pharmacokinetics [11,12]. The creation of NPs, combining drugs with molecular probes, increases the drug half-life in the circulatory system and, specifically, delivers anticancer drugs to target tissues, controlling the drug release through detectors responsive to different stimuli such as pH, temperature, light, ultrasound, and enzymatic activities, thus improving the delivery of the required drug concentration to the area of interest [13,14,15]. By means of improving the circulating half-life, nanomedicines can accumulate in tumors through the enhanced permeability and retention (EPR) effect [16]. Previous studies have reported that EPR varies among mouse models and patients, between tumor types of the same origin, and among tumors and metastases of the same patient, thus explaining the heterogeneous outcomes of NPs clinical trials. To overcome this issue, efforts should focus on methods that increase specific uptake at the tumor site. One strategy is active targeting, in which nanoparticles are conjugated to targeting agents, such as molecules that bind specifically to an overexpressed receptor on target cells [17]. This ligand-receptor interaction increases the accumulation of NPs in the specific cell, inducing their internalization and drug release via receptor-mediated endocytosis [18,19].

Here, we have developed two novel PSMA-targeting liposome platforms. In vitro and in vivo testing was conducted to assess the targeting capabilities of the liposome platform. The efficacy of Talazoparib-loaded liposomes, incorporating the targeting moiety, in combination with BI 2536, was evaluated in a prostate cancer model. Talazoparib, a PARP inhibitor encapsulated in liposomes, has shown activity for the treatment of PCa [20]. Talazoparib inhibits PARP enzymatic activity and regulates DNA damage repair, hence inducing cell death. Liposome-encapsulated Talazoparib shows increased bioavailability, enhanced drug delivery to the target, and reduced side effects [21]. BI 2536 is a PlK1 inhibitor that inhibits tumor growth in vitro and in vivo across various cancers, both via cell cycle arrest, with some safe clinical trials. BI 2536 is often combined with other chemotherapies to enhance its efficacy [22]. While Talazoparib is not currently approved in combination with BI 2536 for prostate cancer (PCa), preclinical studies have shown that BI 2536 exhibits antitumor activity through PLK1 inhibition, leading to G2/M cell-cycle arrest, apoptosis, and attenuation of autophagic flux. However, these findings were demonstrated in neuroblastoma models rather than PCa and do not evaluate combination therapy with Talazoparib. Therefore, additional preclinical studies in prostate cancer are needed to determine whether BI 2536 and Talazoparib have synergistic therapeutic effects [23]. In this study, we investigated a PSMA-targeted liposome platform for specific delivery of Talazoparib to PCa tumors and assessed its efficacy in combination with BI 2536. We believe that targeted delivery of encapsulated Talazoparib enhances drug efficacy and reduces toxicity. We hope this platform enables us to overcome the drug resistance mechanism and improve treatment outcomes.

## 2. Materials and Methods

### 2.1. Reagents

Reagents, including 1-Ethyl-3-(3-dimethylaminopropyl) carbodiimide (EDC), N-Hydroxysuccinimide (NHS), Cholesterol, Matrigel, Triethylamine (TEA), and Trifluoroacetic Acid (TFA), were procured from Sigma-Aldrich (St. Louis, MO, USA). Talazoparib, BI 2536, and DUPA(OtBu)-OH were obtained from MedChemExpress (Monmouth Junction, South Brunswick, NJ, USA). PBS was procured from Cytiva (Marlborough, MA, USA). Additionally, 1,2-distearoyl-sn-glycero-3-phosphoethanolamine-N-[succinyl(polyethylene glycol)-2000] (DSPE-PEG-COOH), 1,2-distearoyl-sn-glycero-3-phosphoethanolamine (DSPE-NH2), and 1,2-Dipalmitoyl-sn-glycero-3-phosphocholine (DPPC) were purchased from Avanti Polar Lipids (Alabaster, AL, USA), HEPES (2-[4-(2-hydroxyethyl)piperazin-1-yl]ethanesulfonic acid) was purchased from Sigma Aldrich (St. Louis, MO, USA). All reagents and solvents were utilized without further purification.

### 2.2. Synthesis

Synthesis of DUPA-DSPE and DUPA-PEG-DSPE was conducted. A quantity of 15 mg of DSPE-NH2 (1,2-distearoyl-sn-glycero-3-phosphoethanolamine) was dissolved in 3 mL of dichloromethane (DCM) and 1.5 mL of methanol (MeOH). Subsequently, 0.98 equivalents of DUPA(OtBu)-OH were dissolved in 200 µL of water and added to the previous mixture. A catalytic amount of triethylamine (TEA) was introduced, and the mixture was stirred for 16 h. The desired product was precipitated using cold diethyl ether. The solid precipitate was then dissolved in 3 mL of trifluoroacetic acid (TFA) and stirred for 3 h. After solvent evaporation, the resulting oil was dissolved in water, frozen, and lyophilized. The final product was obtained as a white solid with a yield of 84%. A similar procedure was employed to synthesize DUPA-PEG-DSPE. In this process, DSPE-NH2 was replaced with DSPE-PEG-NH2 (1,2-distearoyl-sn-glycero-3-phosphoethanolamine-N-[amino(polyethylene glycol)-2000]). The resulting product was washed three times with cold diethyl ether, affording a final yield of 92%.

### 2.3. Liposome Preparation

Liposomes (LIPOs) were created using the thin-layer hydration method. In brief, DPPC, cholesterol, DSPE-PEG, and DSPE-DUPA were dissolved in chloroform in a round-bottom flask at a ratio of 6:3:1:1. The organic solvent was evaporated at 60 °C under reduced pressure to form a thin lipid film, which was left overnight under a hood to eliminate residual solvent. To produce multilamellar liposomes, the lipid film was hydrated with 5 mL of HEPES (20 mM) and then subjected to three cycles (every 3 min) of warming at 60 °C in a water bath and vortexing at 700 rpm. After 30 min at 60 °C, the LIPOs were extruded through polycarbonate membranes with pore sizes of 400, 200, and 100 nm to generate liposomes with varied sizes. For labeling with ^68^Ga and ^64^Cu, 20% *w*/*w* of DSPE-PEG was replaced with DSPE-DOTA, and the same method was used to produce these liposomes. The DOTA-LIPs were resuspended in 3 µL, and 750 mL of DOTA-LIP was conjugated with 300 µL of ^68^Ga or ^64^Cu solution (4 mCi). Excess metal ions were removed via filtration using Amicon 30 KD (Burlington, MA, USA) with 3 PBS washes. Purity was verified using iTLC with saline as the eluent. To prepare Talazoparib-loaded liposomes, 5 mg (dissolved in 50 µL DMSO) was added to the organic phase containing all lipids. The remaining steps followed the previous protocol, and any excess Talazoparib was removed through dialysis against PBS.

### 2.4. LIPOs Characterization

Dynamic light scattering (DLS, Malvern Zetasizer Nano S, Cambridge, MA, USA) was employed to characterize LIPOs’ size, morphology, and zeta potential under hydrated conditions at pH 7.0. The measurements were made with a contact angle of 173°, Backscatter at 25° C. For structural analysis via TEM imaging, liposomes were negatively stained using the following protocol: 5 µL of the sample was absorbed for 1 min onto a carbon-coated grid (EMS, www.emsdiasum.com, order # CF400-CU-(Morgantown, PA, USA)) that had been made hydrophilic with a 20 s glow discharge (25 mA). The adsorption occurred in the presence of osmium vapor to stabilize the liposomes. Excess liquid was removed with filter paper (Whatman #1), and the grid was stained with 1% Uranyl Acetate (EMS catalog # 22400) for 20 s. Grids were examined with a TecnaiG^2^ Spirit BioTWIN (Hillsboro, Oregon, OR, USA), and images were recorded using an AMT NanoSprint43-MkII camera. To determine the talazoparib encapsulation efficiency (EE), the samples were lyophilized, dissolved in acetonitrile/H_2_O (1:1, *v*/*v*), and analyzed by high-performance liquid chromatography (HPLC) (Agilent 1260 Infinity) with a 100 μL sample loop injector. A C18 column (2.1 × 250 mm, 5 μm particle size, Agilent, (Santa Clara, CA, USA) facilitated chromatographic separation. Talazoparib was eluted under isocratic conditions with a binary solvent system [Acetonitrile/H_2_O + 0.1% (*v*/*v*) TFA, 43:57 *v*/*v*], flowing at 1.0 mL/min. EE was determined using the following Equation:

EE (%) = (Talazoparib weight in particles)/(Talazoparib initial feeding amount) × 100

To examine Talazoparib release kinetics, 200 μL of Talazoparib LIPOs solution was placed into Slide-A-Lyzer MINI dialysis microtubes with a 10 kDa molecular cutoff (Thermo Scientific (Boston, MA, USA)) The microtubes were dialyzed against 4 L of PBS buffer (pH 7.4). At each time point, three samples were collected, dried, and then processed. All samples were first destroyed with cold methanol, allowed to dry, and then dissolved in a mixture of acetonitrile and water (1:1, *v*/*v*). They were subsequently analyzed by HPLC to measure talazoparib.

### 2.5. In Vitro Targeting Efficacy

The PCa cell lines (22RV1, PC3, and LNCaP) were sourced from ATCC (Manassas, VA, USA) and thawed for in vitro anticancer testing. 22RV1 and LNCaP cells were grown in RPMI-1640 medium. In contrast, PC3 cells were cultured in F-12K (ATCC) supplemented with 10% fetal bovine serum (Sigma-Aldrich, St. Louis, MO, USA), 1% penicillin (10,000 IU/mL), and streptomycin (10,000 µg/mL). The cells were maintained at 37 °C with 5% CO_2_. Cell viability and counts were assessed using an automated cell counter (Countess, Invitrogen (Boston, MA, USA)) with the trypan blue exclusion method to determine live cells accurately before experiments. Cells were grown and resuspended to a density of 1 × 10^5^ cells/150 µL, using the corresponding growth media, as described before. After pre-wetting the 96-well Filtration Plate MultiScreen^®^ (Millipore Sigma (Burlington, MA, USA)), 150 µL of the cell suspension was loaded into each well. The plates were incubated at 37 °C with 5% CO_2_ for 24 h. Subsequently, the medium was replaced with RPMI-1640 containing the tested formulations at the respective concentrations. Cells and culture medium were initially incubated at 37 °C, after which the cells were cooled to 4 °C before addition of the LIPO formulations. The cells were then incubated with the LIPOs at 4 °C. Following incubation, the cells were washed with cold PBS to remove unbound nanoparticles before further analysis.

### 2.6. In Vitro Cytotoxicity of the LIPOs

In vitro cytotoxicity was assessed for free Talazoparib, BI 2536, empty liposomes, LIPO Talazoparib, and their combinations with 200 nM of BI 2536. Cell viability was measured using an MTT assay (Sigma-Aldrich, St. Louis, MO, USA). Briefly, 22RV1 cells suspended in medium were seeded into 96-well plates at 1 × 10^5^ cells/mL and incubated at 37 °C with 5% CO_2_ for 24 h. The medium was then replaced with RPMI-1640 containing the respective formulations at various concentrations, warmed at 37 °C. After 24, 48, and 72 h, 5.0 mg/mL MTT solution in PBS was added to each well and incubated for 4 h at 37 °C. Media and PBS were then removed, and the formazan crystals formed were dissolved in 200 μL of ethanol per well. Absorbance was measured at 570 nm with a microplate reader (Biotek Cytation 5, Agilent (Santa Clara, CA, USA)). Controls (cells without drug treatment) were set to 100%, and the results from treated cells were expressed as a percentage of the control viability. Each data point was replicated five times.

### 2.7. In Vivo Distribution of the LIPOs

All animal experiments received approval from our Institutional Animal Care and Use Committee (IACUC)/Protocol No. 2013N000141/11-02-2022). The animals were housed in ventilated cages, with unrestricted access to food and water, in an IACUC-certified facility that provides optimal care. A total of 1.5 × 10^6^ 22RV1 cells mixed 1:1 (*v*:*v*) with Matrigel^®^ and PBS (total volume 100 µL) were injected into the left upper flank of naïve nu/nu mice. Once tumors reached a minimum volume of 800 mm^3^, mice with tumors were selected for imaging. LIPOs labeled with ^64^Cu, with or without PSMA tagging, were injected into five naïve nu/nu mice per group. After 24 h, PET and CT scans (micro-PET-SPECT-CT system; Triumph, LABPET, Trifoil) were performed. Following imaging, mice were sacrificed, and their major organs (liver, lungs, heart, spleen, kidneys, intestines, skin, muscle, bone, tumor, blood, and brain) were excised and analyzed for radioactive activity using a γ-counter. The activity data were normalized to the organs’ weight.

### 2.8. Therapeutic Efficacy

1.5 × 10^6^ of 22RV1 cells were injected into the left shoulder of naïve nu/nu mice. The tumors were allowed to grow until reaching 100 mm^3^ before beginning treatment. Tumor size was measured using a digital caliper (Traceable^®^ Digital Caliper, Stainless Steel, 6in (Webster, TX, USA)). Mice were randomly assigned to different treatment groups. In all cases, the agent was administered intravenously three days on days 0, 3, 6, 9, 12, 15, and 18. Tumor growth was monitored using a digital caliper until animals reached the predefined humane endpoint or became moribund. All mice were euthanized when they or when tumor size exceeded 1500 mm^3^. Survival was tracked and analyzed using the Kaplan–Meier method. until animals became moribund. Mice were randomly assigned to different treatment groups. In all cases, the agent was administered intravenously every 3 days for up to 21 days, for a total of 7 injections. Tumor growth was monitored using a digital caliper. All mice were euthanized when they died or when tumor size exceeded 1500 mm^3^. Survival was tracked and analyzed using the Kaplan–Meier method.

### 2.9. Statistical Analysis

All statistical analyses were conducted using GraphPad Prism 10.1.2 (Boston, MA, USA) and SPSS 29.0.1 software (Armonk, NY, USA). Experiments were performed in triplicate, and variables were presented as mean ± SD. For tumor growth curves, statistical significance was evaluated by two-way ANOVA followed by Tukey’s post hoc test. *** *p* < 0.001. Kaplan–Meier survival curves were analyzed using the log-rank (Mantel–Cox) test. For pairwise survival comparisons, *p* values were adjusted for multiple comparisons using the Holm method.

## 3. Results

### 3.1. Synthesis and Physicochemical Characterization of PSMA-Targeted Liposomes

**Figure 1:**
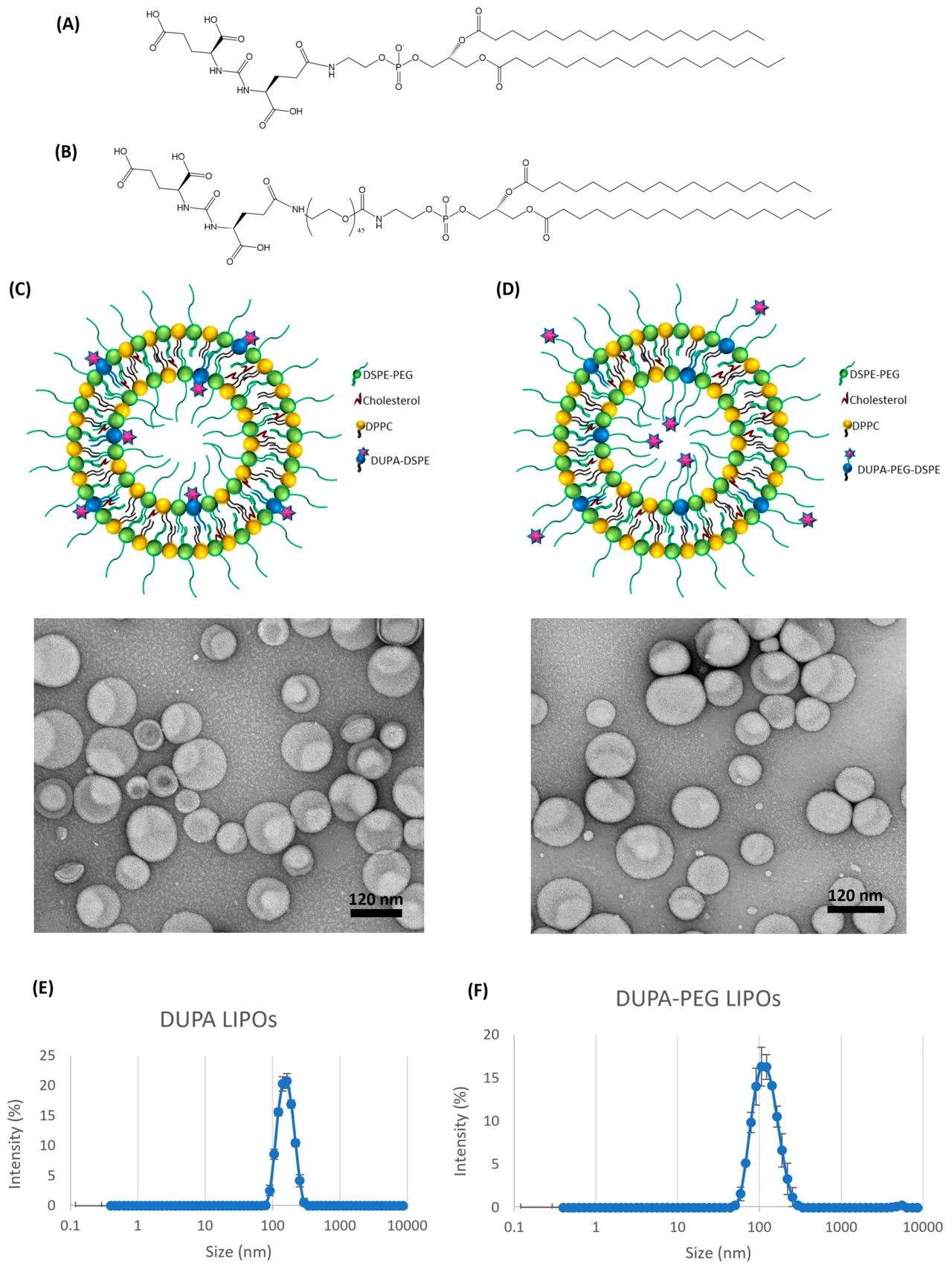
Schematic Representation of the monomers and particles prepared. Chemical structures of the two lipid-based DUPA compounds modified with DSPE (1,2-distearoyl-sn-glycero-3-phosphoethanolamine) (**A**) and DSPE-PEG (1,2-distearoyl-sn-glycero-3-phosphoethanolamine-N-[amino(polyethylene glycol)-2000]) (**B**). Schematic representations and TEM images of the liposomes produced with DUPA-DSPE (**C**) and with DUPA-PEG-DSPE (**D**). Scale bar: 120 nm. Hydrodynamic diameter of DUPA LIPOs (**E**) and DUPA-PEG LIPOs (**F**) via dynamic light scattering analysis.

To enable PSMA-directed drug delivery, two targeting lipids were synthesized by conjugating the PSMA ligand DUPA either directly to DSPE (DUPA-DSPE) or through a PEG2000 spacer (DUPA-PEG-DSPE) (Figure 1A,B). Both conjugates were successfully synthesized with high isolated yields and confirmed by spectroscopic characterization (Supplementary Figures S1 and S2). The final isolated yields were 84% for DUPA-DSPE and 92% for DUPA-PEG-DSPE. Targeted liposomes were prepared by thin-film hydration followed by membrane extrusion to obtain relatively uniform-sized vesicles [24]. Transmission electron microscopy (TEM) confirmed the formation of spherical vesicles with well-defined morphology (Figure 1C,D). Dynamic light scattering (DLS) measurements demonstrated that DUPA-DSPE liposomes exhibited a mean hydrodynamic diameter of approximately 150 nm, whereas DUPA-PEG-DSPE liposomes displayed a slightly smaller average diameter of approximately 120 nm (Figure 1E,F). Both formulations exhibited narrow size distributions (PDI < 0.2), indicating relatively homogeneous particle populations.

**Figure 2:**
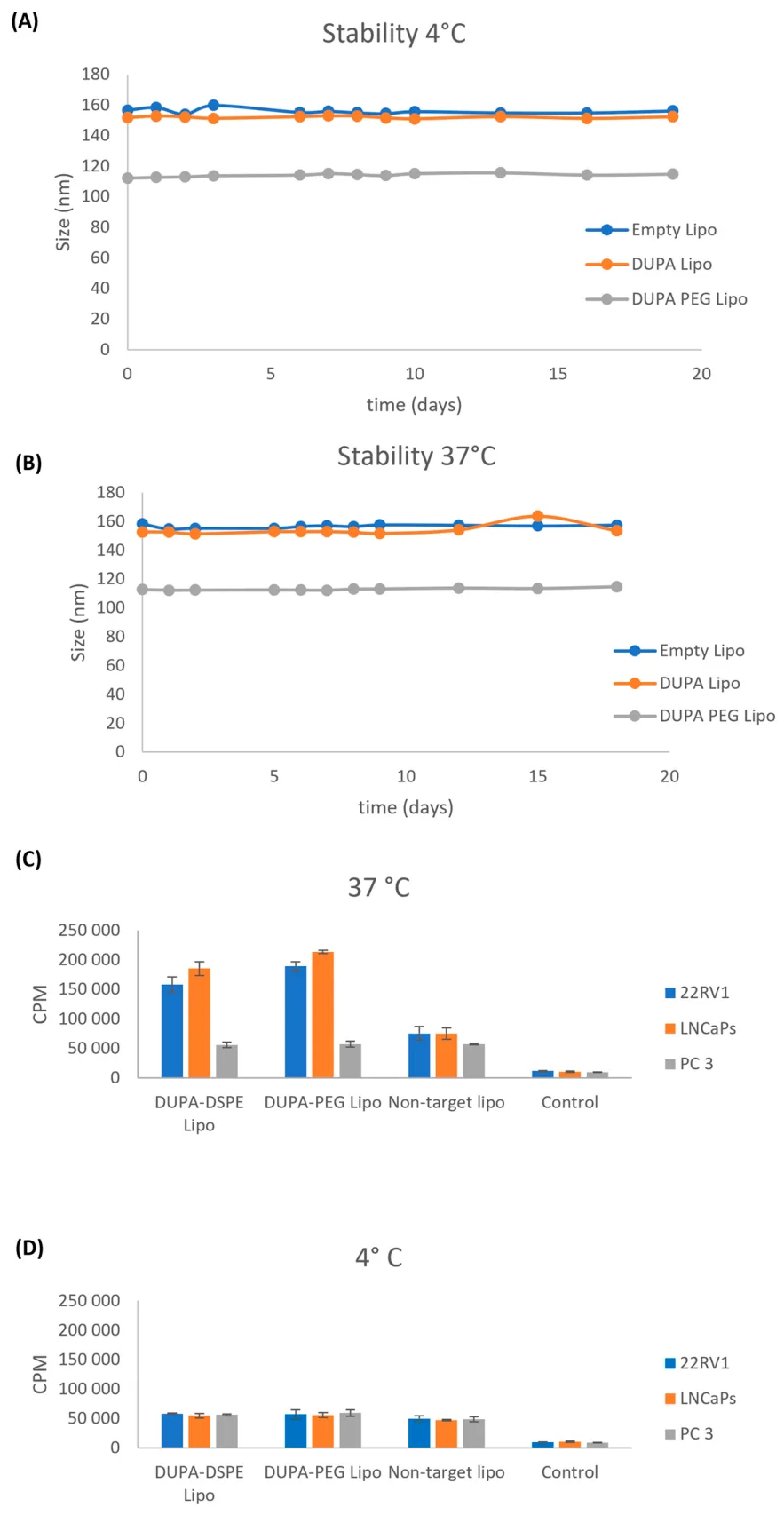
Stability of LIPOs, DUPA LIPOs, and DUPA-PEG. LIPO formulations at 4 °C (**A**) and 37 °C (**B**). Comparison study of accumulation of DUPA-DSPE liposome, DUPA-PEG Liposome, and non-targeted liposomes in PCa cell lines (22Rv1, LNCaP, and PC3) at 37 °C (**C**) and at 4 °C (**D**).

The colloidal stability of targeted and non-targeted liposomes was evaluated during storage at 4 °C and under physiological conditions (37 °C). No significant changes in particle size or PDI were observed during the storage period (Figure 2A,B), demonstrating that incorporation of the targeting ligands did not adversely affect nanoparticle stability.

To evaluate the influence of ligand presentation on PSMA recognition, both targeted formulations were compared with non-targeted liposomes using prostate cancer cell lines exhibiting high (LNCaP), intermediate (22Rv1), and low (PC3) PSMA expression. Radiolabeled DUPA-PEG liposomes demonstrated the highest cellular association with PSMA-positive cells, whereas non-targeted liposomes exhibited comparable accumulation across all cell lines (Figure 2C). Cellular association increased approximately three-fold in LNCaP cells compared with PC3 cells (*p* < 0.05), confirming PSMA-dependent targeting. DUPA-PEG liposomes consistently exhibited greater cellular association than DUPA-DSPE liposomes, suggesting that presentation of DUPA through a PEG spacer improves ligand accessibility for receptor recognition.

To differentiate receptor-mediated internalization from nonspecific surface binding, uptake studies were repeated at 4 °C (Figure 2D). Under these conditions, cellular association was markedly reduced across all formulations, consistent with inhibition of energy-dependent endocytic pathways. The residual signal likely reflects nonspecific liposome adsorption to the cell surface [24,25].

#### Characterization of Talazoparib-Loaded PSMA-Targeted Liposomes

Following identification of DUPA-PEG as the lead targeting formulation, talazoparib-loaded liposomes were prepared for therapeutic evaluation (Figure 4A). TEM analysis demonstrated that drug loading did not alter liposome morphology, while DLS measurements confirmed a mean particle diameter of approximately 130 nm, comparable to that of unloaded formulations (Figure 4B and Supplementary Figure S3). The encapsulation efficiency was approximately 85%, indicating efficient incorporation of talazoparib into the liposomal formulation. Storage stability at 4 °C showed no significant changes in particle size during the observation period. Under physiological conditions (37 °C), only modest changes in particle diameter were observed over time (Figure 4B) and (Supplementary Table S2), suggesting acceptable colloidal stability during the anticipated therapeutic window. Drug release studies demonstrated a biphasic release profile (Figure 4C). Approximately 40% of the encapsulated talazoparib was released during the first 10 h, followed by a slower, sustained release phase, reaching approximately 50% cumulative release after 72 h. This sustained release profile may support prolonged drug exposure and potentially contribute to increased intratumoral talazoparib concentrations following administration.

### 3.2. In Vivo Biodistribution of PSMA-Targeted Liposomes

**Figure 3:**
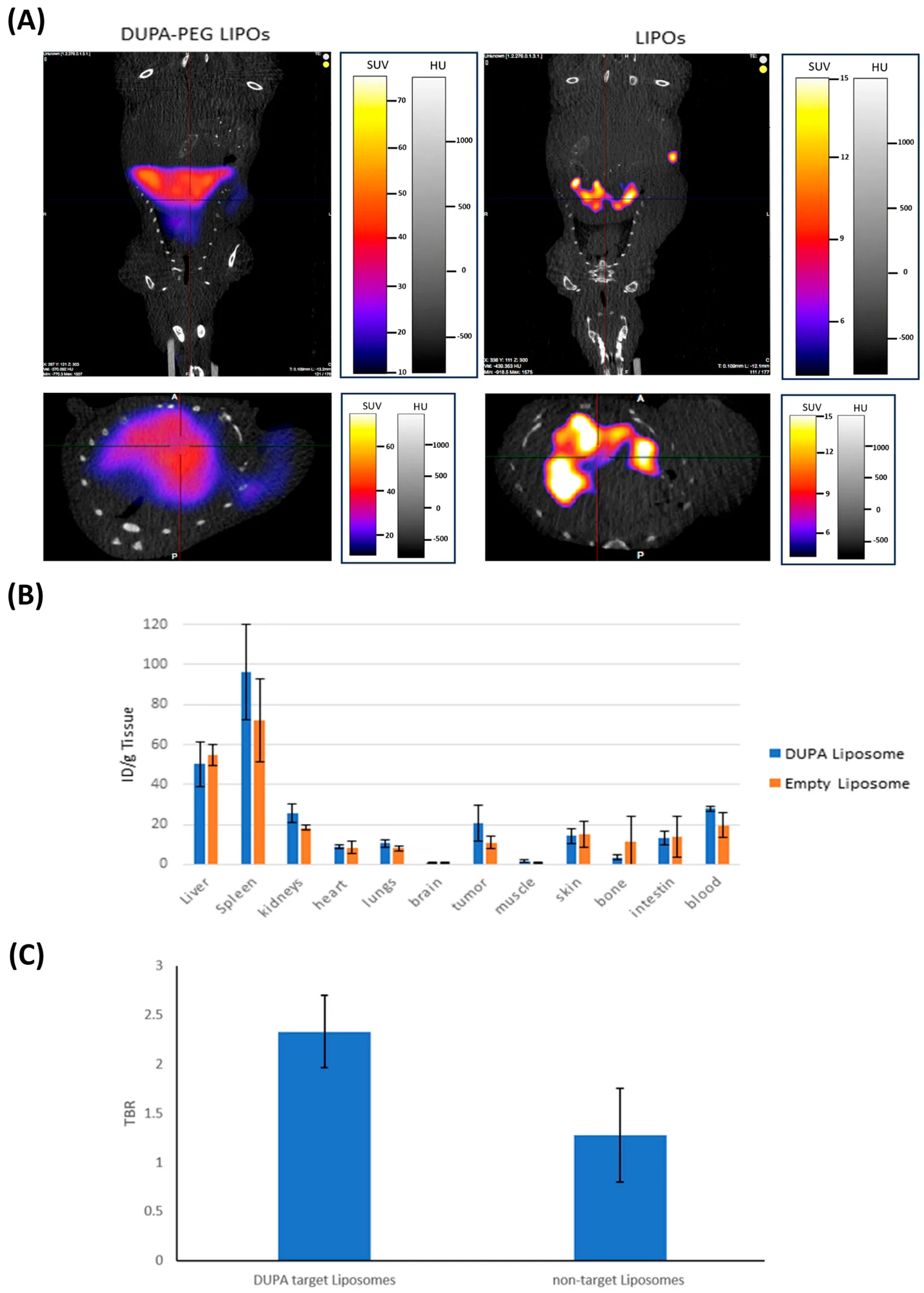
^64^Cu imaging. PET/CT analysis of ^64^Cu accumulation on subcutaneous PCa xenografts (22Rv1 cells), at 24 h post systemic administration of DUPA-PEG-LIPOs vs. LIPOs, respectively (**A**). Biodistribution in mice bearing subcutaneous PCa xenografts (22Rv1 cells), at 24 h post systemic administration of DUPA-PEG-LIPOs vs. LIPOs. Data are presented as the mean ± SD; *n* = 5 mice per experimental group (**B**). Tumor-to-blood ratio (TBR) of ^64^Cu-labeled DUPA-PEG-LIPOs and LIPOs at 24 h (**C**).

The biodistribution of targeted and non-targeted liposomes was investigated in 22Rv1tumor-bearing mice using ^64^Cu-labeled formulations. PET/CT imaging performed 24 h after intravenous administration demonstrated predominant accumulation within the liver for both formulations, consistent with uptake by the mononuclear phagocyte system (Figure 3A). Quantitative biodistribution analysis confirmed high liver accumulation (approximately 45–50%ID/g) together with substantial uptake in the spleen and kidneys (Figure 3B). Blood activity remained detectable after 24 h, indicating prolonged systemic circulation of both formulations.

Importantly, DUPA-PEG liposomes exhibited significantly greater tumor accumulation than non-targeted liposomes (approximately 20 versus 11%ID/g, *p* < 0.05). Consequently, the tumor-to-blood ratio increased from approximately 1.3 for non-targeted liposomes to approximately 2.3 for DUPA-PEG liposomes (Figure 3C), indicating improved tumor selectivity.

No statistically significant differences were observed between the two formulations in the accumulation within major healthy organs, suggesting that incorporation of the targeting ligand primarily enhanced tumor localization without substantially altering systemic biodistribution.

### 3.3. Cell Viability Studies

**Figure 4:**
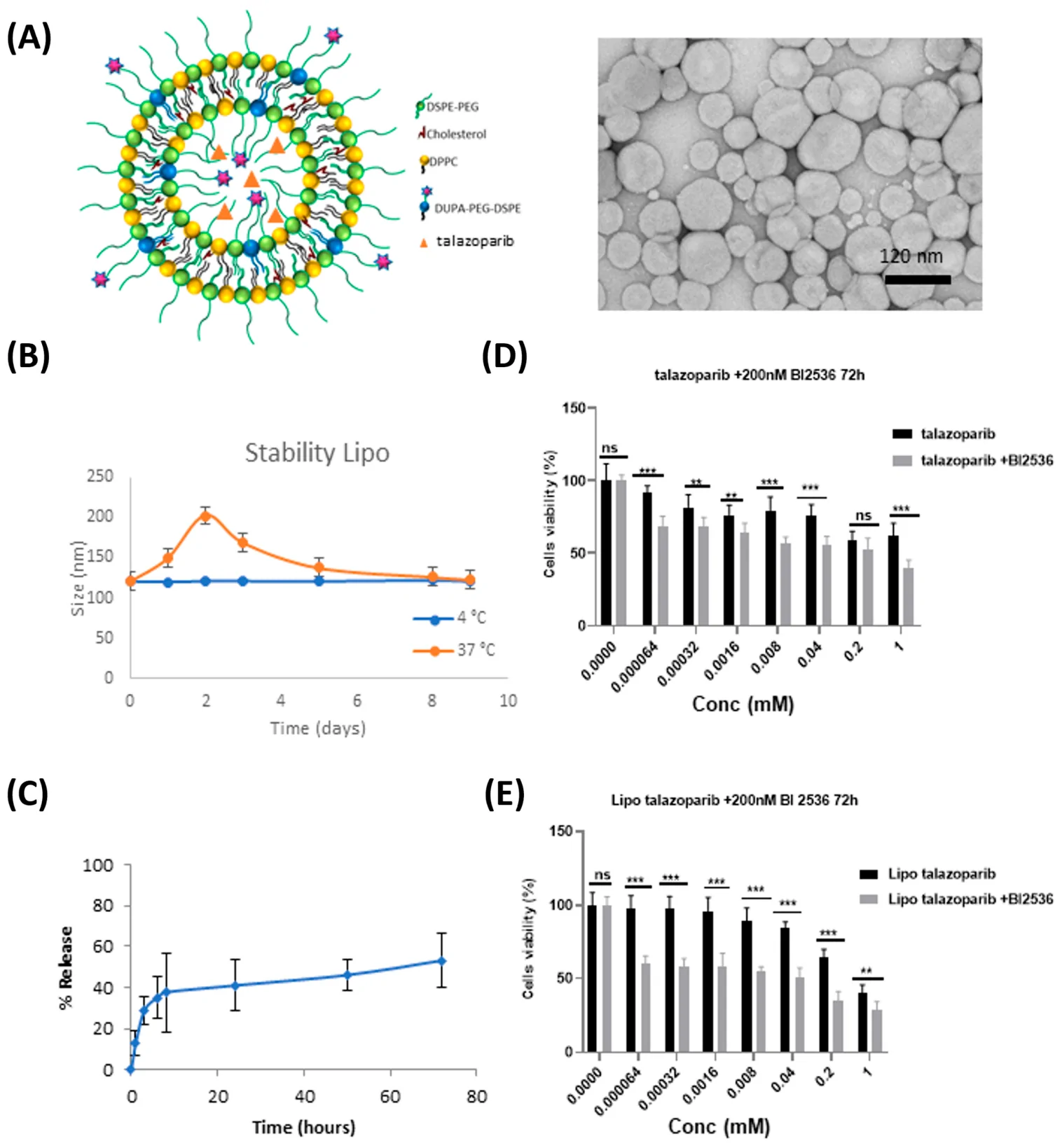
Characterization and stability of liposomes with TZPB-DUPA and BI 2536. Schematic representations and TEM image of the liposomes produced with TZPB-DUPA LIPOs (**A**). Stability of TZPB-DUPA LIPOs formulation at 37 °C and 4 °C (**B**). Talazoparib release kinetics profiles from liposomes (dialysis performed against 4 L PBS). Results are expressed as the average ± SD (*n* = 6) (**C**). Viability of 22Rv1 incubated with TZPB or the combination of TZPB and BI2536 (**D**) and the TZPB-DUPA LIPOs or TZPB-DUPA LIPOs in combination with BI 2536 at 72 h (**E**). Statistical significance was determined by two-way ANOVA, followed by Sidak’s multiple-comparisons test. ns, not significant; ** *p* < 0.01, and *** *p* < 0.001.

Using the MTT assay, the cytotoxic activity of LIPOs and drugs was assessed in the 22RV1 PCa cell line at 24 h, 48 h, and 72 h (Figure 4D,E and Supplementary Figure S4 and Supplementary Table S3). Talazoparib concentrations ranging from 60 nM to 1 mM were tested for TZPB- and TZPB-DUPA-loaded LIPOs. As shown in Supplementary Figure S4, cell viability decreased in a dose-dependent manner at all evaluated time points (24 h, 48 h, and 72 h). TZPB and TZPB-DUPA LIPOs exhibited IC_50_ values of 320 and 280 µM at 72 h, respectively. The cytotoxicity of empty LIPOs was also assessed in 22RV1 cells, showing cell viability above 80% at all tested time points and lipid concentrations ranging from 32 nM to 50 mM. The relatively high IC_50_ values observed for TZPB are consistent with previously reported values. Based on these results, a combination therapy, TZPB and BI 2536, was investigated. These findings support the potential of combining BI 2536 and TZPB for PCa treatment. In 22RV1 Cells, BI 2536 exhibited an IC_50_ of 400 nM at 72 h. For the in vitro combination study, a non-toxic concentration of BI 2536 (200 nM) was selected. The interaction between talazoparib and BI 2536 was quantitatively evaluated using the Chou–Talalay combination index (CI) and Bliss independence model based on the 72 h cytotoxicity data in 22Rv1 cells. Both analyses demonstrated a concentration-dependent interaction between the two agents (Supplementary Figure S4 and Table S3). At lower concentrations of talazoparib, the combination with BI 2536 showed a clear synergistic interaction. At higher concentrations of talazoparib, the magnitude of synergy was reduced, with the interaction ranging from weak synergy to predominantly additive effects. Overall, these findings indicate that the interaction between talazoparib and BI 2536 was concentration-dependent, with the strongest synergistic effects observed at lower talazoparib concentrations.

### 3.4. Therapeutic Effect on a Prostate Cancer Model

**Figure 5:**
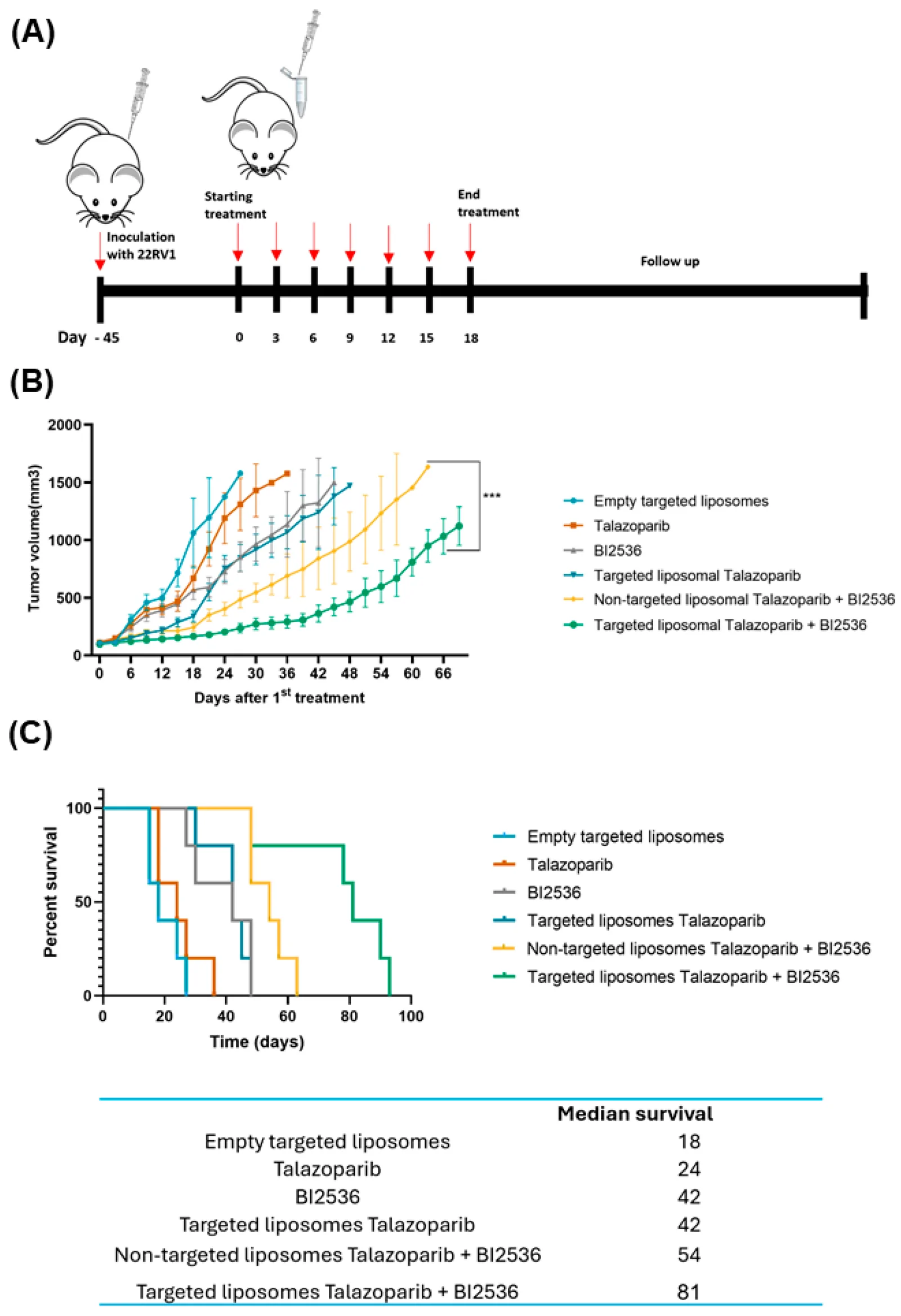
Therapeutic effect on a prostate cancer model. Schematic representations of preclinical therapeutic efficacy on 22Rv1 xenograft model (**A**). Variation in tumor size over time for all experimental groups (average *n* = 5 mice) (Data are presented as mean ± SEM. Statistical significance was evaluated by two-way ANOVA followed by Tukey’s post hoc test. *** *p* < 0.001 comparing Targeted liposomal talazoparib + BI 2536 against Non-targeted liposomal talazoparib + BI 2536 and control/monotherapy groups) (**B**); Kaplan–Meier survival curves and list of median survivals (**C**). Table summarizing the median survival (days) for the different therapeutic groups (**C**).

The therapeutic efficacy of lipid nanoparticles (LIPOs) encapsulating TZPB was evaluated using a murine 22Rv1 xenograft model (Figure 5). Figure 5A illustrates the experimental timeline. We administered the following concentrations of talazoparib 1 mg/Kg (body weight) with a concentration of BI 2536 at 0.7 µg/mL. The study included six treatment groups, with each group receiving intravenous injections every three days for 18 days, for a total of seven injections on days 0, 3, 6, 9, 12, 15, and 18. The treatment groups were free TZPB, free BI 2536, target liposomes containing TZPB, non-target liposomes containing TZPB combined with BI 2536, target liposomes containing TZPB combined with BI 2536, and empty target liposomes as the control group. Tumor growth was monitored following initiation of treatment, as shown in Figure 5B.

At baseline, no statistically significant differences in mean tumor volume were observed among the treatment groups. The empty target liposome control group showed progressive tumor growth throughout the study. Mice treated with free TZPB exhibited a modest delay in tumor growth compared with the control group, indicating limited therapeutic activity of free TZPB in this model. Incorporation of TZPB into target liposomes further delayed tumor growth compared with free TZPB, suggesting improved therapeutic activity following liposomal formulation. Free BI 2536 exhibited a therapeutic effect comparable to that of target liposomes containing TZPB. The combination of non-target liposomal TZPB with BI 2536 and, particularly, the combination of target liposomal TZPB with BI 2536 produced greater tumor growth suppression than either monotherapy. Tumor growth remained suppressed during the treatment period, followed by tumor regrowth after treatment cessation. Notably, the target liposomal TZPB plus BI 2536 group maintained tumor growth suppression for the longest period, with tumor regrowth becoming more apparent after approximately day 40.

Overall survival was evaluated using Kaplan–Meier analysis (Figure 5C). The median survival times were 18 days for mice treated with empty target liposomes, 24 days for free TZPB, 42 days for free BI2536, 42 days for target liposomes containing TZPB, 54 days for non-target liposomal TZPB combined with BI 2536, and 81 days for target liposomal TZPB combined with BI 2536 (Supplementary Table S1). The targeted talazoparib-loaded liposome + BI2536 group exhibited the longest median survival (81 days), compared with 54 days for the corresponding non-targeted combination. Although this difference was significant in the unadjusted pairwise log-rank analysis (*p* = 0.0211), it did not remain statistically significant following Holm correction for multiple comparisons (adjusted *p* = 0.0872). Therefore, while the targeted combination showed a numerical prolongation of survival, the present study does not provide definitive statistical evidence of a targeting-specific survival advantage in the combination setting. These findings demonstrate enhanced therapeutic efficacy of the targeted liposomal TZPB formulation when combined with BI 2536.

## 4. Discussion

Targeted drug delivery has emerged as a promising strategy to improve the therapeutic index of anticancer agents by enhancing drug accumulation at the tumor site while limiting systemic exposure. Among the various molecular targets investigated for prostate cancer, prostate-specific membrane antigen (PSMA) remains one of the most attractive because of its high expression in advanced, metastatic, and castration-resistant prostate cancers, combined with its ability to undergo rapid receptor-mediated internalization following ligand binding. These characteristics make PSMA an ideal target for nanoparticle-mediated delivery of therapeutic payloads [25,26].

In the present study, we developed two novel PSMA-targeted liposomal formulations by conjugating the small-molecule ligand DUPA either directly to DSPE or through a PEG2000 spacer. The enhanced cellular association observed for DUPA-functionalized liposomes is consistent with the well-established biology of prostate-specific membrane antigen (PSMA), which undergoes constitutive receptor-mediated internalization following ligand binding. PSMA is internalized predominantly through clathrin-mediated endocytosis via a conserved MXXXL internalization motif located within its cytoplasmic domain. After ligand binding, the receptor-ligand complex is rapidly transported into early endosomes and subsequently trafficked through late endosomal and lysosomal compartments, where receptor recycling and intracellular processing occur. This efficient internalization pathway has been extensively exploited for the targeted delivery of imaging probes, radionuclides, antibody-drug conjugates, and nanoparticle-based therapeutics in prostate cancer because it promotes intracellular accumulation of the therapeutic cargo while maintaining high tumor selectivity [27,28,29].

For liposomal formulations, receptor-mediated uptake represents only the initial step in intracellular drug delivery. Following endocytosis, liposomes are exposed to the progressively acidic environment of endosomes and lysosomes, where phospholipid membrane destabilization and enzymatic degradation can facilitate release of the encapsulated drug. Although classical phospholipid liposomes are generally not specifically engineered for efficient endosomal escape, partial destabilization of the vesicle membrane during endosomal maturation may increase intracellular availability of hydrophobic payloads such as talazoparib.

Although both formulations demonstrated selective association with PSMA-expressing cells, the PEG-linked formulation consistently exhibited greater cellular association than the directly conjugated lipid. This finding highlights the importance of ligand presentation on the nanoparticle surface [30,31,32]. Direct conjugation positions the targeting ligand close to the phospholipid bilayer, where steric hindrance may partially limit receptor accessibility. In contrast, incorporation of a PEG spacer extends the ligand beyond the hydrated PEG corona, facilitating receptor recognition while maintaining the stealth properties conferred by PEGylation. Similar improvements in receptor accessibility following PEG-mediated ligand presentation have been reported for several actively targeted liposomal systems, supporting the rationale for selecting DUPA-PEG as the lead formulation for subsequent therapeutic studies.

The improved therapeutic activity observed for the DUPA-PEG liposomes is likely attributable to the combined effects of enhanced cellular internalization through PSMA-mediated endocytosis and subsequent intracellular release of talazoparib following endosomal and lysosomal processing. Nevertheless, direct visualization of intracellular trafficking and quantification of endosomal escape were beyond the scope of the present study and therefore warrant further investigation using fluorescence colocalization imaging and mechanistic intracellular trafficking assays.

The physicochemical characteristics of the developed liposomes were consistent with those generally considered favorable for systemic drug delivery. Both targeted formulations exhibited narrow size distributions and excellent colloidal stability under storage and physiological conditions. Particle diameters of approximately 120–150 nm fall within the range commonly reported for long-circulating liposomal formulations and are expected to support prolonged blood circulation while permitting passive accumulation within tumors through the enhanced permeability and retention (EPR) effect. Importantly, incorporation of the targeting ligand did not adversely affect nanoparticle stability, suggesting that active targeting can be achieved without compromising formulation robustness.

The in vitro targeting studies further demonstrated that nanoparticle association correlated with PSMA expression levels, supporting receptor-specific recognition of the DUPA-functionalized liposomes. Although experiments performed at 4 °C cannot completely exclude nonspecific membrane interactions, the marked reduction in nanoparticle uptake under conditions that inhibit energy-dependent endocytosis strongly suggests that cellular internalization primarily occurs through receptor-mediated mechanisms. Following ligand binding, PSMA is known to undergo clathrin-mediated endocytosis, leading to trafficking through early and late endosomal compartments before lysosomal processing. Such intracellular trafficking is expected to facilitate release of the encapsulated drug within tumor cells, thereby increasing intracellular drug availability compared with passive extracellular drug diffusion. While these intracellular events were not directly investigated in the present study, they provide a plausible mechanistic explanation for the enhanced therapeutic activity observed for the targeted formulation.

The biodistribution profile obtained by PET/CT imaging demonstrated that both targeted and non-targeted liposomes followed the characteristic pharmacokinetic behavior of long-circulating PEGylated liposomes after systemic administration. At 24 h post-injection, the highest accumulation was observed in the liver (approximately 45–50%ID/g), followed by the spleen and kidneys, reflecting uptake by the mononuclear phagocyte system (MPS). This clearance pathway is expected for nanoparticles within the 100–150 nm size range and is primarily mediated by Kupffer cells in the liver and macrophages residing in the spleen, which efficiently recognize circulating nanoparticles despite PEGylation. Although PEGylation significantly prolongs blood circulation by reducing protein adsorption and opsonization, complete evasion of MPS uptake remains difficult to achieve, particularly for phospholipid-based nanoparticles of this size [33,34]. Despite substantial hepatic accumulation, the targeted DUPA-PEG liposomes exhibited significantly greater tumor accumulation than the non-targeted formulation (approximately 20% versus 11%ID/g at 24 h), representing nearly a two-fold improvement in tumor localization. More importantly, the tumor-to-blood ratio (TBR) increased from approximately 1.3 for non-targeted liposomes to 2.3 for DUPA-PEG liposomes, indicating that active targeting enhanced selective tumor retention beyond that achieved through passive accumulation alone. Since both formulations displayed comparable blood activity after 24 h, the higher TBR observed for DUPA-PEG liposomes is unlikely to result solely from prolonged circulation and instead suggests improved interaction with PSMA-expressing tumor cells following extravasation into the tumor microenvironment.

These findings are consistent with the current understanding that nanoparticle accumulation within tumors is governed by two sequential processes. Initially, long-circulating nanoparticles accumulate within tumor tissue through the enhanced permeability and retention (EPR) effect, which depends primarily on nanoparticle size, circulation half-life, and tumor vascular permeability. Subsequently, active targeting ligands contribute by promoting selective binding and cellular internalization rather than substantially increasing the total quantity of nanoparticles reaching the tumor. Consequently, active targeting often produces only modest increases in total tumor accumulation (typically 1.5- to 3-fold) while generating considerably greater improvements in intracellular delivery and therapeutic efficacy [18,35]. The approximately two-fold increase in tumor uptake observed for the DUPA-PEG formulation therefore falls within the range commonly reported for receptor-targeted liposomal systems and is biologically meaningful.

Comparison with previously reported PSMA-targeted liposomal formulations further supports the performance of the present system. Patil et al. demonstrated enhanced uptake of folate-conjugated liposomes targeting PSMA-expressing prostate cancer cells, although quantitative in vivo tumor accumulation was not extensively characterized. Similarly, Wong et al. reported that anti-PSMA scFv-functionalized liposomes exhibited approximately two-fold greater tumor accumulation than non-targeted liposomes, together with improved PET imaging contrast in prostate tumor xenografts. The tumor uptake and TBR values obtained in the present study are therefore consistent with previously reported actively targeted PSMA-directed nanocarriers while demonstrating that the relatively simple DUPA-PEG lipid architecture can achieve comparable targeting efficiency without requiring antibody conjugation [29,36].

It should be emphasized, however, that the biodistribution study evaluated radiolabeled liposomes rather than direct tissue concentrations of talazoparib. Consequently, although increased nanoparticle accumulation strongly suggests improved tumor delivery of the encapsulated drug, direct quantification of intratumoral talazoparib concentrations will be required to establish the relationship between carrier accumulation and local drug exposure.

Talazoparib was selected as the therapeutic payload because of its potent inhibition of poly(ADP-ribose) polymerase (PARP), an essential enzyme involved in DNA damage repair [37,38]. Despite its clinical efficacy in tumors harboring homologous recombination deficiency, talazoparib exhibits poor aqueous solubility and dose-limiting systemic toxicity, both of which may restrict its therapeutic window. Liposomal encapsulation therefore represents an attractive strategy to improve its pharmacokinetic profile while facilitating selective tumor delivery. The sustained release profile observed in the present study may further contribute to maintaining therapeutic drug concentrations over an extended period following tumor accumulation. Although formal mathematical modeling of release kinetics was beyond the scope of this work, the observed biphasic release profile is consistent with an initial diffusion-controlled release of surface-associated drug followed by slower release from the lipid bilayer, as commonly reported for liposomal formulations of hydrophobic anticancer agents.

The rationale for combining talazoparib with the PLK1 inhibitor BI 2536 is supported by the complementary biological functions of these two therapeutic targets [39]. PARP inhibition promotes accumulation of DNA damage by impairing single-strand break repair, whereas PLK1 inhibition disrupts mitotic progression and further compromises DNA damage repair pathways. Previous preclinical studies have demonstrated that inhibition of PLK1 enhances sensitivity to PARP inhibitors through increased genomic instability and mitotic catastrophe, thereby providing a mechanistic basis for combination therapy. In the present study, co-administration of BI 2536 enhanced the antitumor activity of talazoparib-loaded liposomes both in vitro and in vivo. Although the targeted talazoparib-loaded liposome + BI2536 group exhibited the longest median survival observed in the study (81 days), compared with 54 days for the corresponding non-targeted combination, this difference did not remain statistically significant after correction for multiple comparisons. Thus, the survival data suggest a potential benefit associated with PSMA-targeted delivery but do not establish a statistically significant targeting-specific survival advantage. The small group size (*n* = 5) further limits the statistical power of this comparison, and larger, appropriately powered studies will be required to determine whether the observed numerical survival difference reflects a reproducible therapeutic benefit of PSMA targeting. Future studies incorporating quantitative synergy analyses, together with mechanistic investigations of DNA damage signaling and apoptosis, will further clarify the molecular interactions underlying this combination. Compared with previously reported PSMA-targeted liposomal systems, the present formulation demonstrates several favorable characteristics, including particle sizes within the optimal range for systemic administration, high encapsulation efficiency, prolonged colloidal stability, and improved tumor accumulation relative to non-targeted controls. Moreover, the direct comparison between DUPA-DSPE and DUPA-PEG formulations provides important insight into the influence of ligand presentation on targeting efficiency, an aspect that has received relatively limited attention in previous PSMA-targeted liposomal studies. These findings may therefore contribute to the rational design of future actively targeted liposomal formulations for prostate cancer.

Several limitations of this study should be acknowledged. First, direct pharmacokinetic quantification of talazoparib within tumor tissue was not performed, preventing definitive conclusions regarding local drug concentrations. Second, mechanistic studies investigating apoptosis, DNA damage signaling, PLK1 inhibition, and intracellular drug trafficking were beyond the scope of the present work. Third, additional control groups, including free talazoparib combined with BI 2536 and non-targeted talazoparib-loaded liposomes administered as monotherapy, would further strengthen mechanistic interpretation of the therapeutic studies. Finally, the relatively small number of animals per treatment group reflects the proof-of-concept nature of this investigation, and larger preclinical studies incorporating comprehensive safety, pharmacokinetic, and toxicological evaluations will be required before clinical translation can be considered. Despite these limitations, the present study demonstrates that rational engineering of ligand presentation on the liposomal surface can substantially improve PSMA-targeted drug delivery. Together, these findings support continued development of DUPA-functionalized liposomes as a versatile platform for targeted delivery of molecular therapeutics in prostate cancer.

## 5. Conclusions

We developed two novel PSMA-targeted liposomal formulations based on DUPA-functionalized phospholipids and demonstrated that presentation of the targeting ligand through a PEG spacer significantly improved cellular association with PSMA-expressing prostate cancer cells compared with direct lipid conjugation. The optimized DUPA-PEG formulation exhibited favorable physicochemical properties, excellent colloidal stability, selective tumor targeting, and enhanced tumor accumulation relative to non-targeted liposomes.

Encapsulation of talazoparib within the targeted liposomes improved antitumor activity compared with the free drug, while combination treatment with BI 2536 further delayed tumor progression and prolonged survival in a prostate cancer xenograft model. Additional pharmacokinetic and mechanistic studies are needed to directly correlate nanoparticle biodistribution with intratumoral drug concentrations and to define the molecular basis of the enhanced therapeutic response. PSMA-targeted liposomes demonstrated enhanced tumor localization and, when combined with BI2536, produced the longest median survival observed in this proof-of-concept study. However, the survival difference between targeted and non-targeted combination therapy did not remain statistically significant after correction for multiple comparisons, and larger studies are required to establish a targeting-specific survival benefit. More broadly, the modular DUPA-functionalized liposomal platform described here may be readily adapted for the delivery of other therapeutic payloads or combination regimens targeting PSMA-positive malignancies, supporting its further development as a versatile nanomedicine platform for prostate cancer.

## Data Availability

The raw data of this study were generated at the Center for Precision Imaging, Division of Nuclear Medicine and Molecular Imaging, Department of Radiology, and Wellman Center for Photomedicine, Massachusetts General Hospital, and are available upon request to the corresponding author.

## Supplementary Materials

The following supporting information can be downloaded at: https://www.mdpi.com/article/10.3390/pharmaceutics18091150/s1, Figure S1: Schematic representation of the Synthesis of DUPA-DSPE and NMR characterization.; Figure S2: Schematic representation of the Synthesis of DUPA-PEG-DSPE and NMR characterization.; Figure S3: Hydrodynamic diameter and Zeta potential of DOTA-DUPA LPNs (A) and DOTA-DUPA-PEG LNPs (B) via dynamic light scattering analysis; Figure S4: Viability of 22RV1 incubated with TZPB, TZPB+BI 2536, TZPB-DUPA LNPs, TZPB-DUPA LNPs + BI 2563, BI 2536 and LNPs at 3 different time points; Table S1: Kaplan–Meier survival analysis of the 22Rv1 xenograft study; Table S2: Physicochemical characterization; Table S3: Quantitative drug-interaction analysis talazoparib (μM) and BI 2536 200 (nM).
