# Supplementary material for: PSMA-Targeted Liposomes for Therapeutic Delivery into a Prostate Cancer Cell Model
Source: Pharmaceutics. 2026 Sep 14;18(9):1150. doi: 10.3390/pharmaceutics18091150 (PMC13610626; doi:10.3390/pharmaceutics18091150)
Supplement: Supplementary file 1 [file pharmaceutics-18-01150-s001.zip › pharmaceutics-4428101-supplementary.pdf]

Synthesis and characterization of DUPA-DSPE

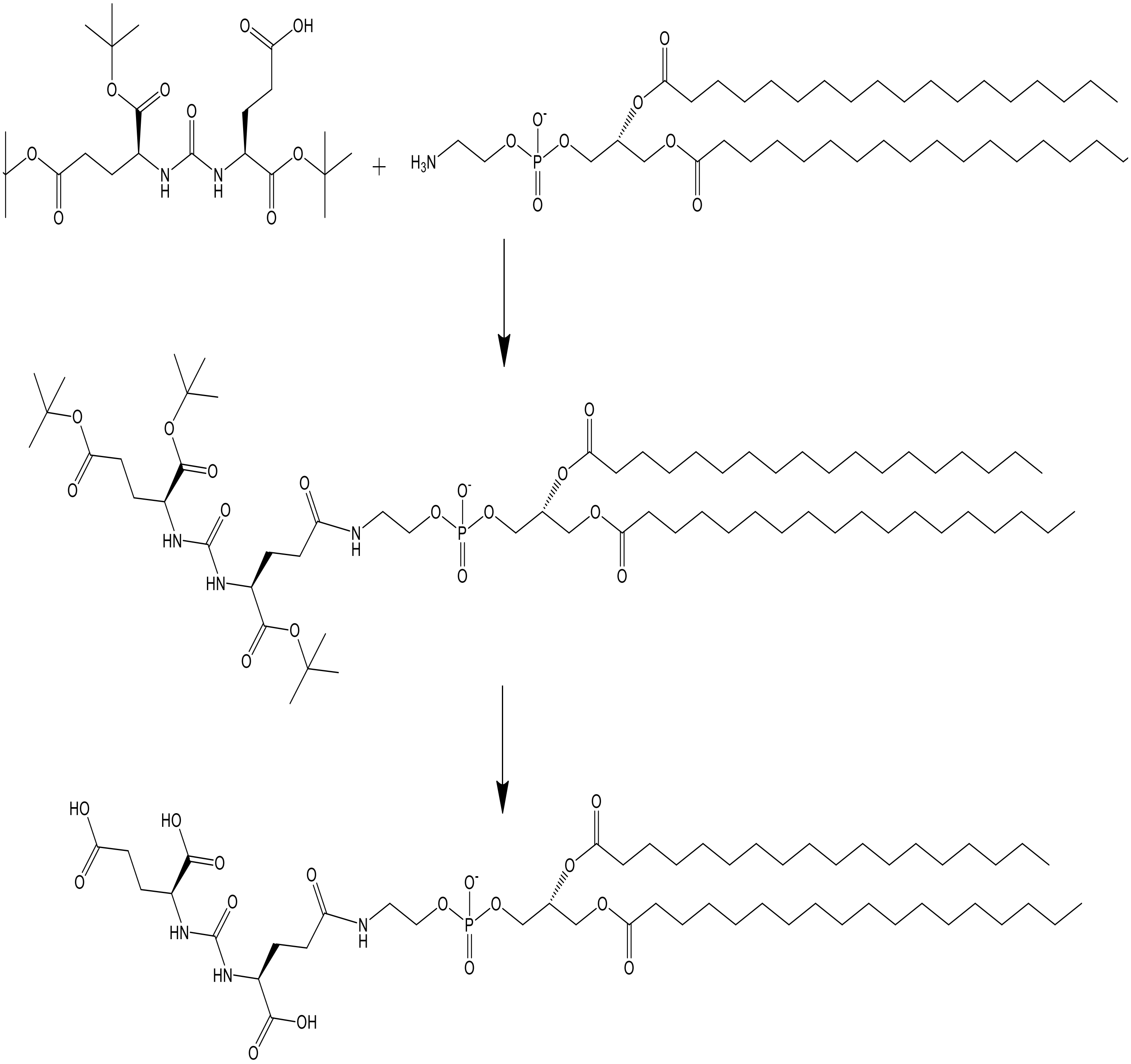

Chemical Formula: $C_{52}H_{95}N_3O_{16}P^-$

Exact Mass: 1048,65

Molecular Weight: 1049,30

m/z: 1048.64 (100.0%), 1049.65 (57.9%), 1050.65 (20.4%), 1051.66 (3.1%), 1051.65 (2.1%), 1049.64 (1.1%)

Elemental Analysis: C, 59.52; H, 9.13; N, 4.00; O, 24.40; P, 2.95

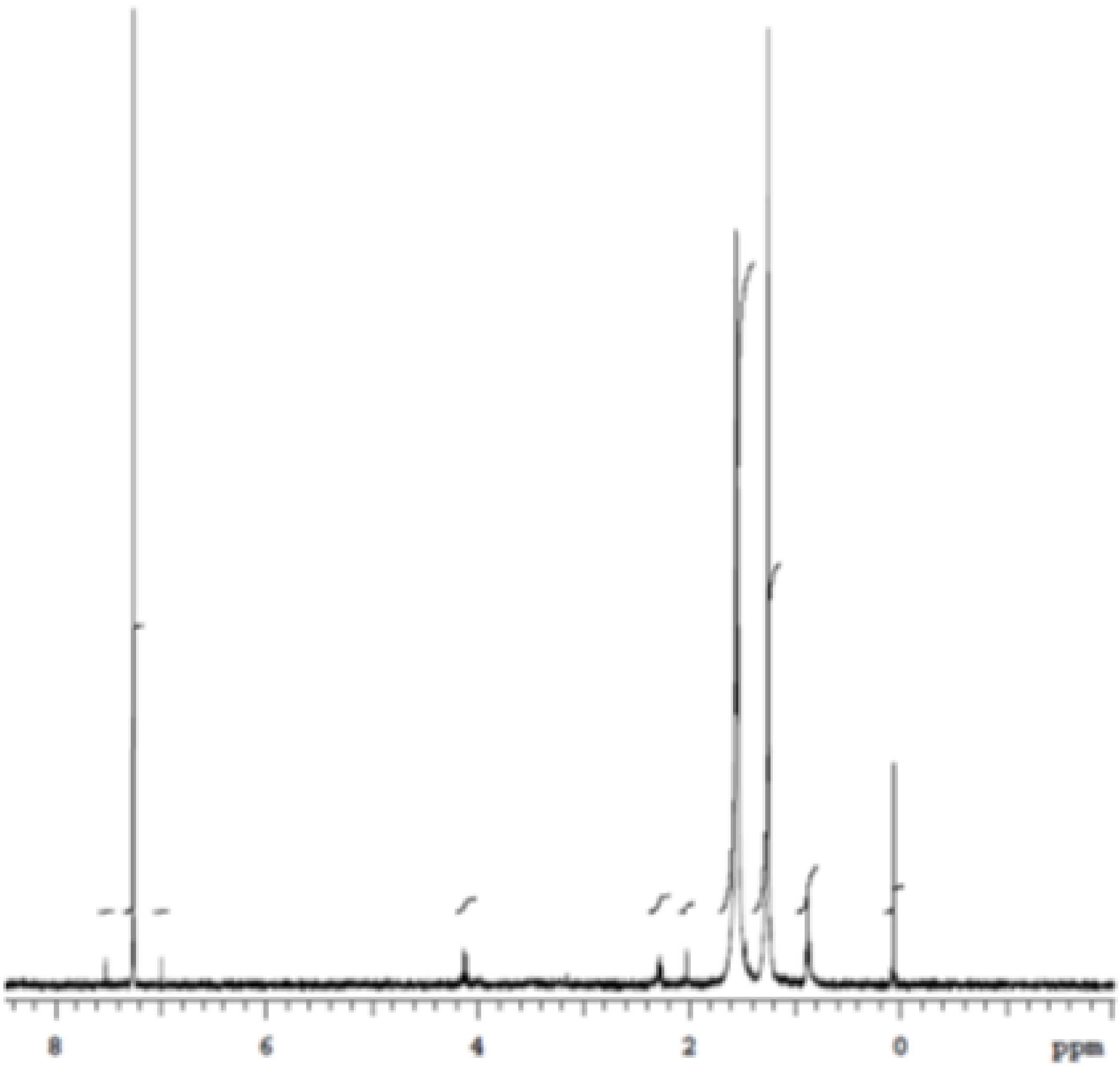

Supplementary Figure S1. Schematic representation of the Synthesis of DUPA-DSPE and NMR characterization.

Synthesis and characterization of DUPA-PEG-DSPE

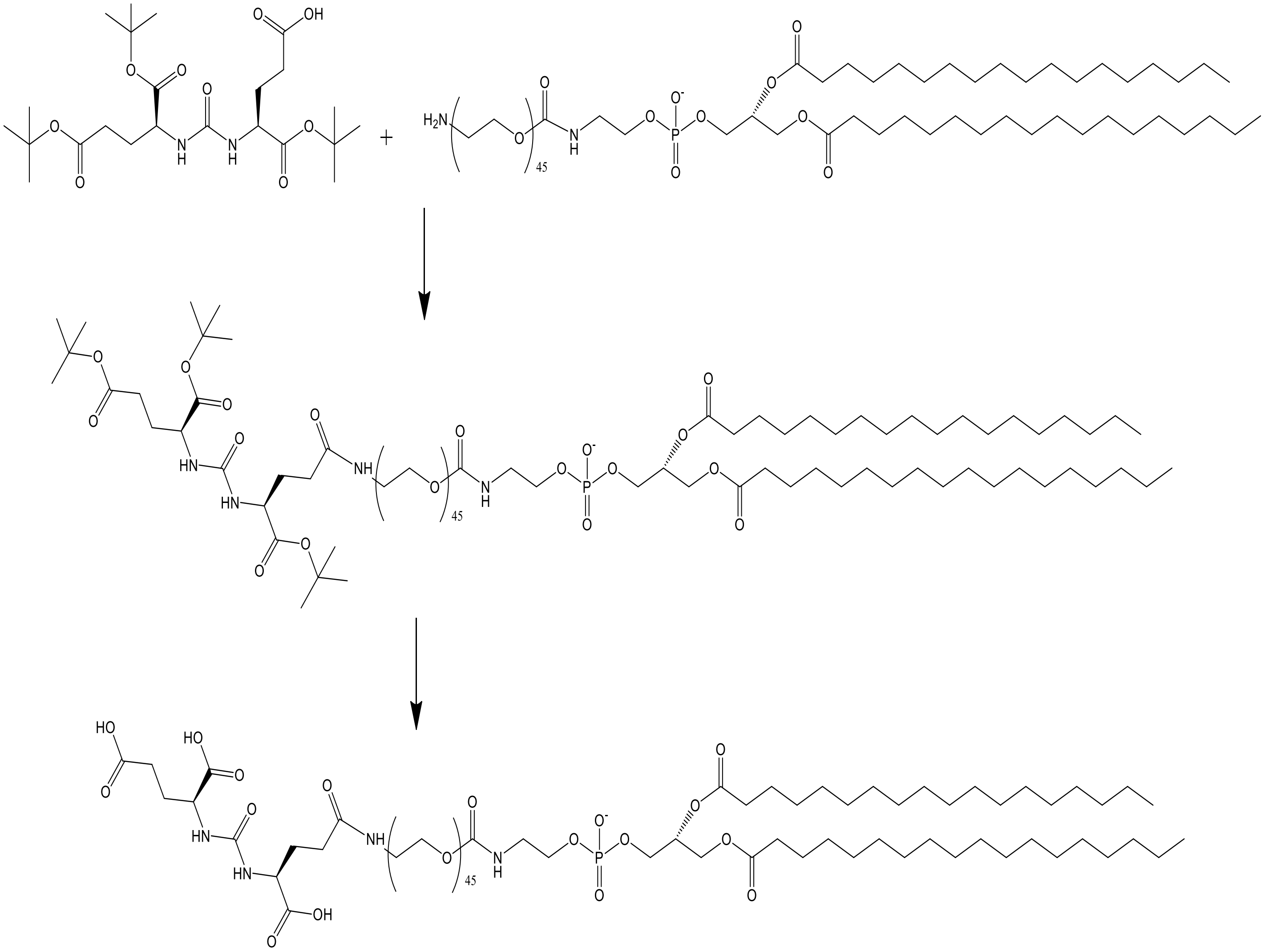

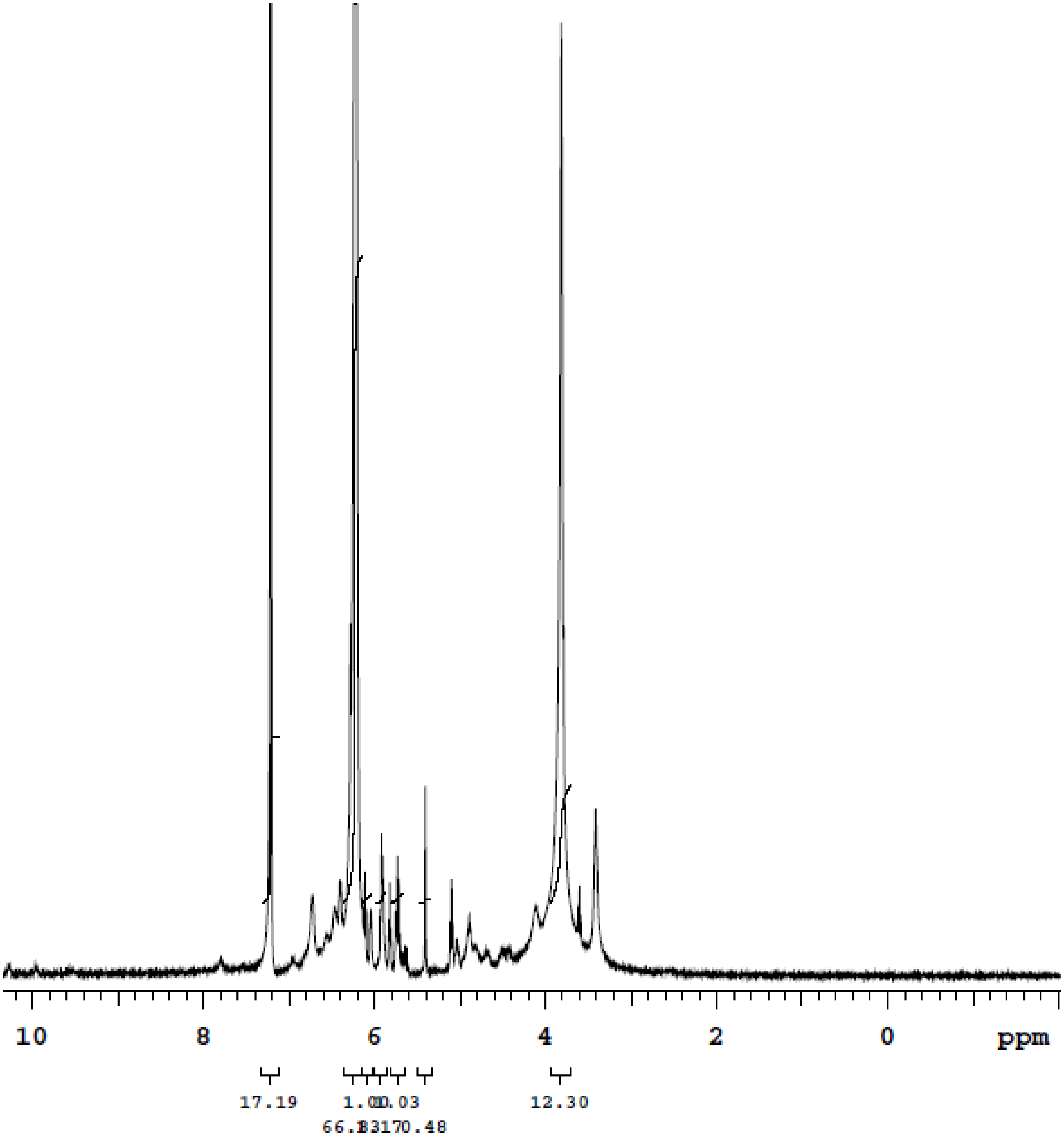

Supplementary Figure S2. Schematic representation of the Synthesis of DUPA-PEG-DSPE and NMR characterization.

A)

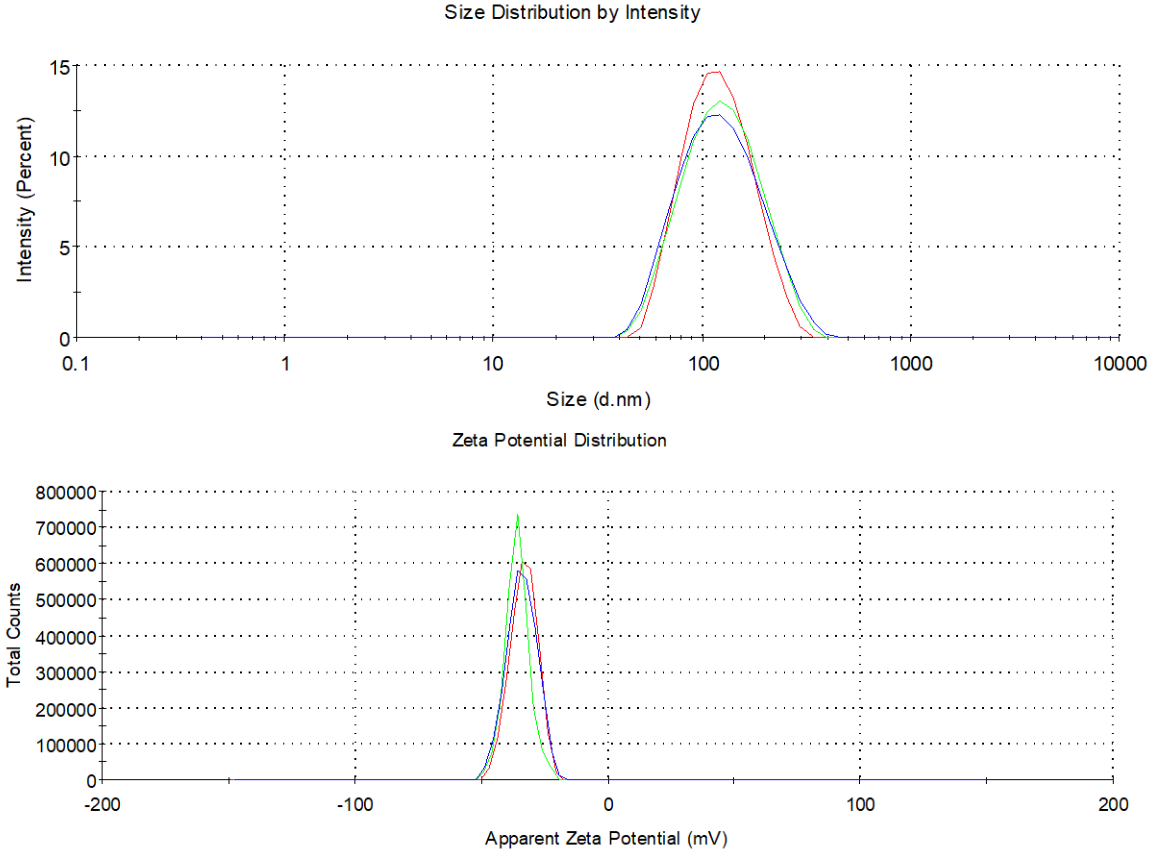
Size Distribution by Intensity
Intensity (Percent)
15
10
5
0
0.1
1
10
100
1000
10000
Size (d.nm)
Zeta Potential Distribution
Total Counts
800000
700000
600000
500000
400000
300000
200000
100000
0
-200
-100
0
100
200
Apparent Zeta Potential (mV)

B)

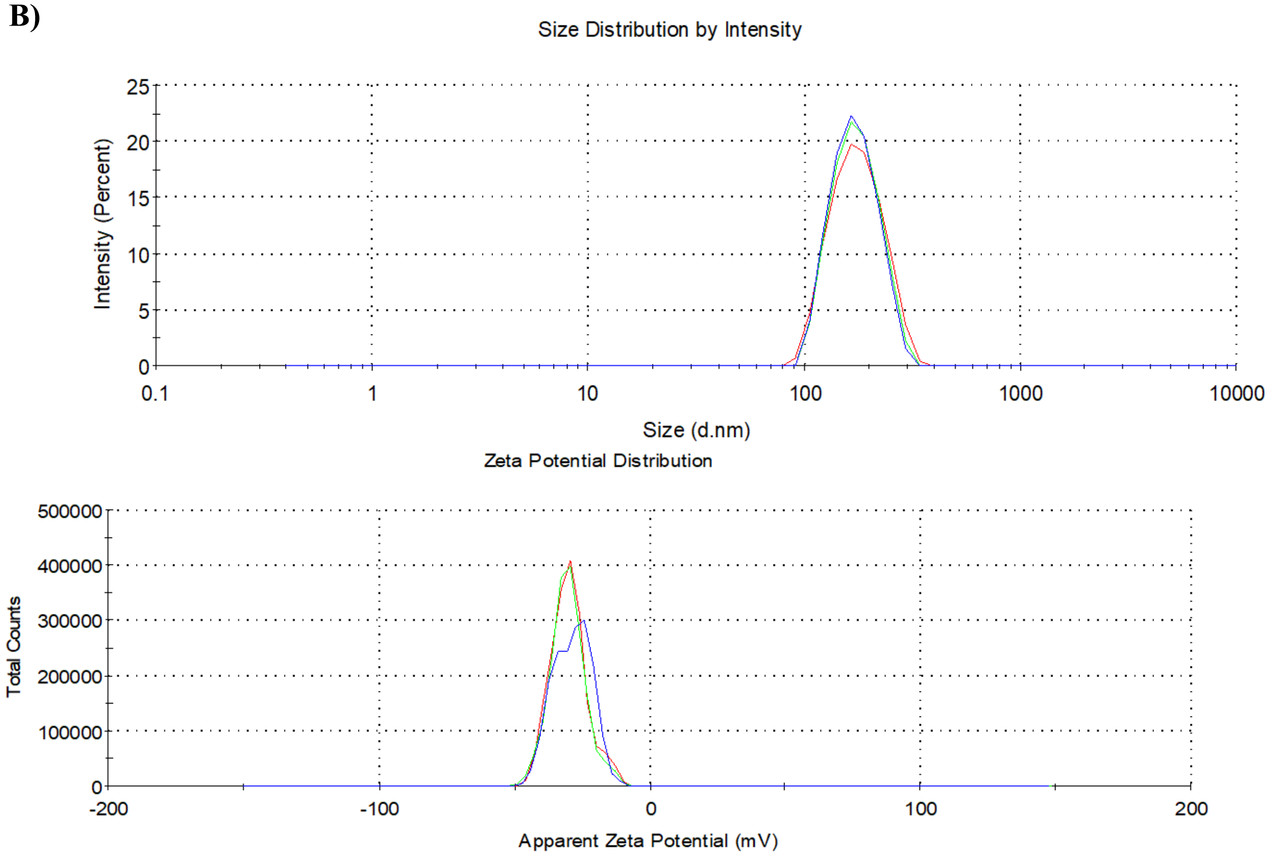
Size Distribution by Intensity
Intensity (Percent)
25
20
15
10
5
0
0.1
1
10
100
1000
10000
Size (d.nm)
Zeta Potential Distribution
Total Counts
500000
400000
300000
200000
100000
0
-200
-100
0
100
200
Apparent Zeta Potential (mV)

Supplementary Figure S3. Hydrodynamic diameter and Zeta potential of DOTA-DUPA LPNs (A) and DOTA-DUPA-PEG LNPs (B) via dynamic light scattering analysis.

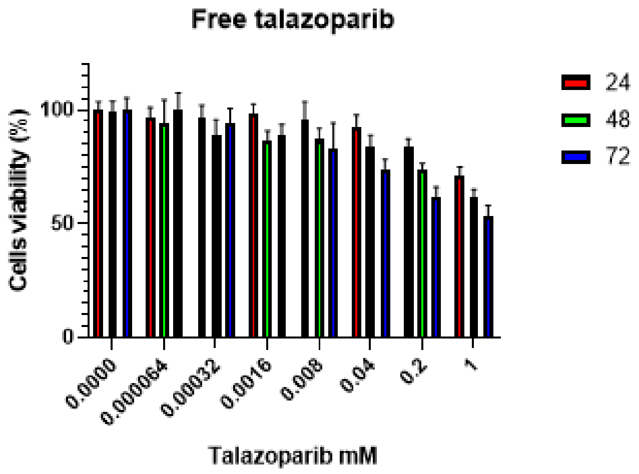
Free talazoparib
Cells viability (%)
0
50
100
0.0000
0.000064
0.00032
0.0016
0.008
0.04
0.2
1
Talazoparib mM
24
48
72

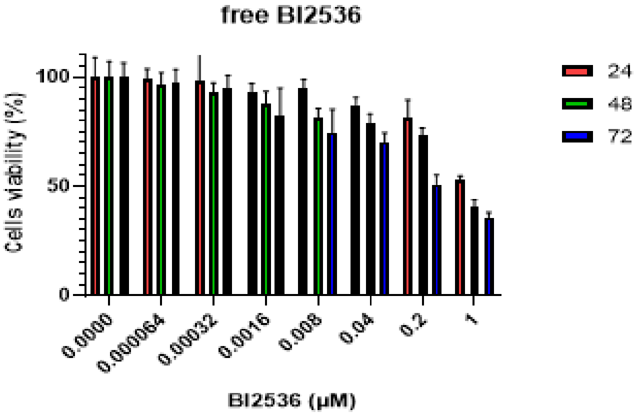
free BI2536
Cells viability (%)
0
50
100
0.0000
0.000064
0.00032
0.0016
0.008
0.04
0.2
1
BI2536 (μM)
24
48
72

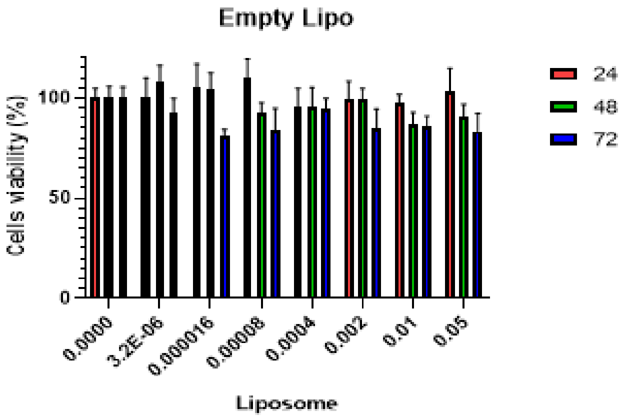
Empty Lipo
Cells viability (%)
0
50
100
0.0000
3.2E-06
0.000016
0.00008
0.0004
0.002
0.01
0.05
Liposome
24
48
72

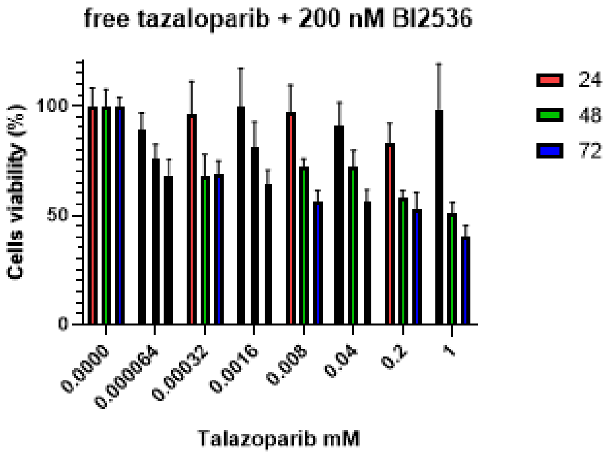
free tazaloparib + 200 nM BI2536
Cells viability (%)
0
50
100
0.0000
0.000064
0.00032
0.0016
0.008
0.04
0.2
1
Talazoparib mM
24
48
72

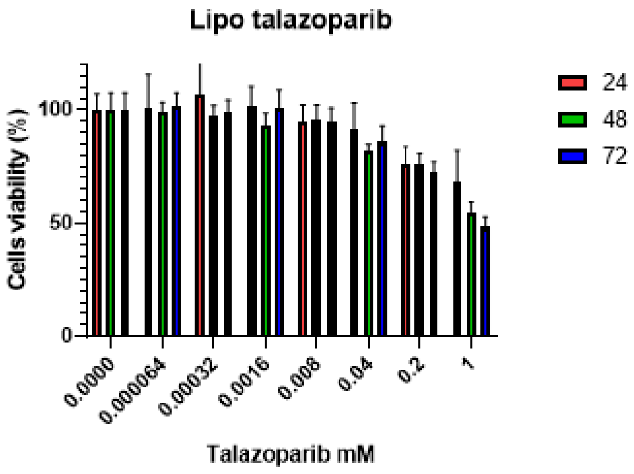
Lipo talazoparib
Cells viability (%)
0
50
100
0.0000
0.000064
0.00032
0.0016
0.008
0.04
0.2
1
Talazoparib mM
24
48
72

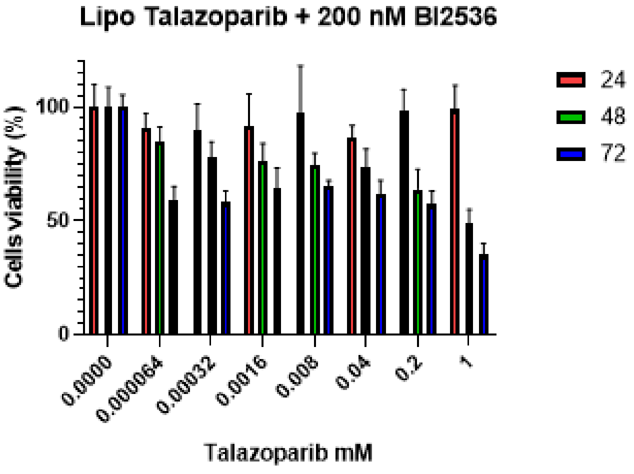
Lipo Talazoparib + 200 nM BI2536
Cells viability (%)
0
50
100
0.0000
0.000064
0.00032
0.0016
0.008
0.04
0.2
1
Talazoparib mM
24
48
72

Supplementary Figure S4. Viability of 22RV1 incubated with TZPB, TZPB+BI 2536, TZPB-DUPA LNPs, TZPB-DUPA LNPs +BI2563, BI2536 and LNPs at 3 different time points.

Table S1. Kaplan–Meier survival analysis of the 22Rv1 xenograft study

Treatment group	n	Individual survival times (days)	Median survival (days)
Empty targeted liposomes	5	15, 15, 18, 24, 27	18
Free talazoparib	5	18, 18, 24, 27, 36	24
BI2536	5	27, 30, 42, 48, 48	42
Targeted liposomal talazoparib	5	30, 42, 42, 45, 48	42
Non-targeted liposomal talazoparib + BI2536	5	48, 48, 54, 57, 63	54
Targeted liposomal talazoparib + BI2536	5	48, 78, 81, 90, 93	81

**Log-rank (Mantel-Cox) test (recommended)**	
Chi square	45.52
df	5
P value	<0.0001
P value summary	****
Are the survival curves sig different?	Yes

Comparison	Log-rank p	Holm-adjusted p
Empty vs. talazoparib	0.2547	0.5094
Empty vs. BI2536	0.0042	0.0424
Empty vs. targeted liposomal talazoparib	0.0017	0.0255
Empty vs. non-targeted combination	0.0017	0.0255

Empty vs. targeted combination	0.0017	0.0255
Talazoparib vs. BI2536	0.0259	0.0872
Talazoparib vs. targeted liposomal talazoparib	0.0062	0.0500
Talazoparib vs. non-targeted combination	0.0017	0.0255
Talazoparib vs. targeted combination	0.0017	0.0255
BI2536 vs. targeted liposomal talazoparib	0.8836	0.8836
BI2536 vs. non-targeted combination	0.0174	0.0872
BI2536 vs. targeted combination	0.0082	0.0562
Targeted liposomal talazoparib vs. non-targeted combination	0.0080	0.0562
Targeted liposomal talazoparib vs. targeted combination	0.0045	0.0424
Non-targeted combination vs. targeted combination	0.0211	0.0872

Table S2. Physicochemical characterization

Parameter	Value
Size	120 ± 5 nm
PDI	0.12 ± 0.02
Encapsulation Efficiency	85%
Drug Loading	
Storage Stability	20 days
Batch variation	CV <5%

Table S3. Quantitative drug-interaction analysis talazoparib (µM) and BI2536 200 (nM)

Talazoparib (µM)	Fa	Chou–Talalay CI	CI interpretation	Bliss score	Bliss interpretation
0.000064	0.318	91.77	Antagonism	+20.80	Synergy
0.00032	0.311	112.13	Antagonism	+3.76	Weak synergy
0.0016	0.358	32.22	Antagonism	-12.72	Antagonism
0.008	0.434	5.22	Antagonism	-5.52	Antagonism
0.04	0.442	5.71	Antagonism	-4.41	Near additive
0.2	0.472	6.52	Antagonism	-4.58	Near additive
1	0.598	1.60	Mild antagonism	-3.44	Near additive
